# Supplementary material for: Transcription factor-driven coordination of cell cycle exit and lineage-specification in vivo during granulocytic differentiation: In memoriam Professor Niels Borregaard
Source: Nat Commun. 2022 Jun 23;13:3595. doi: 10.1038/s41467-022-31332-1 (PMC9225994; doi:10.1038/s41467-022-31332-1)
Supplement: Supplementary file 1 — Supplementary Information [file 41467_2022_31332_MOESM1_ESM.pdf]

Transcription factor-driven coordination of cell cycle exit and lineage-specification *in vivo* during granulocytic differentiation

In memoriam Prof. Niels Borregaard

Kim Theilgaard-Mönch*[#], Sachin Pundhir[#], Kristian Reckzeh, Jinyu Su, Marta Tapia, Benjamin Furtwängler, Johan Jendholm, Janus Schou Jakobsen, Marie Sigurd Hasemann, Kasper Jermiin Knudsen, Jack Bernard Cowland, Anna Fossum, Erwin Schoof, Mikkel Bruhn Schuster, Bo T Porse*

[#]equal contribution

*Corresponding author

Supplementary Figures and Legends

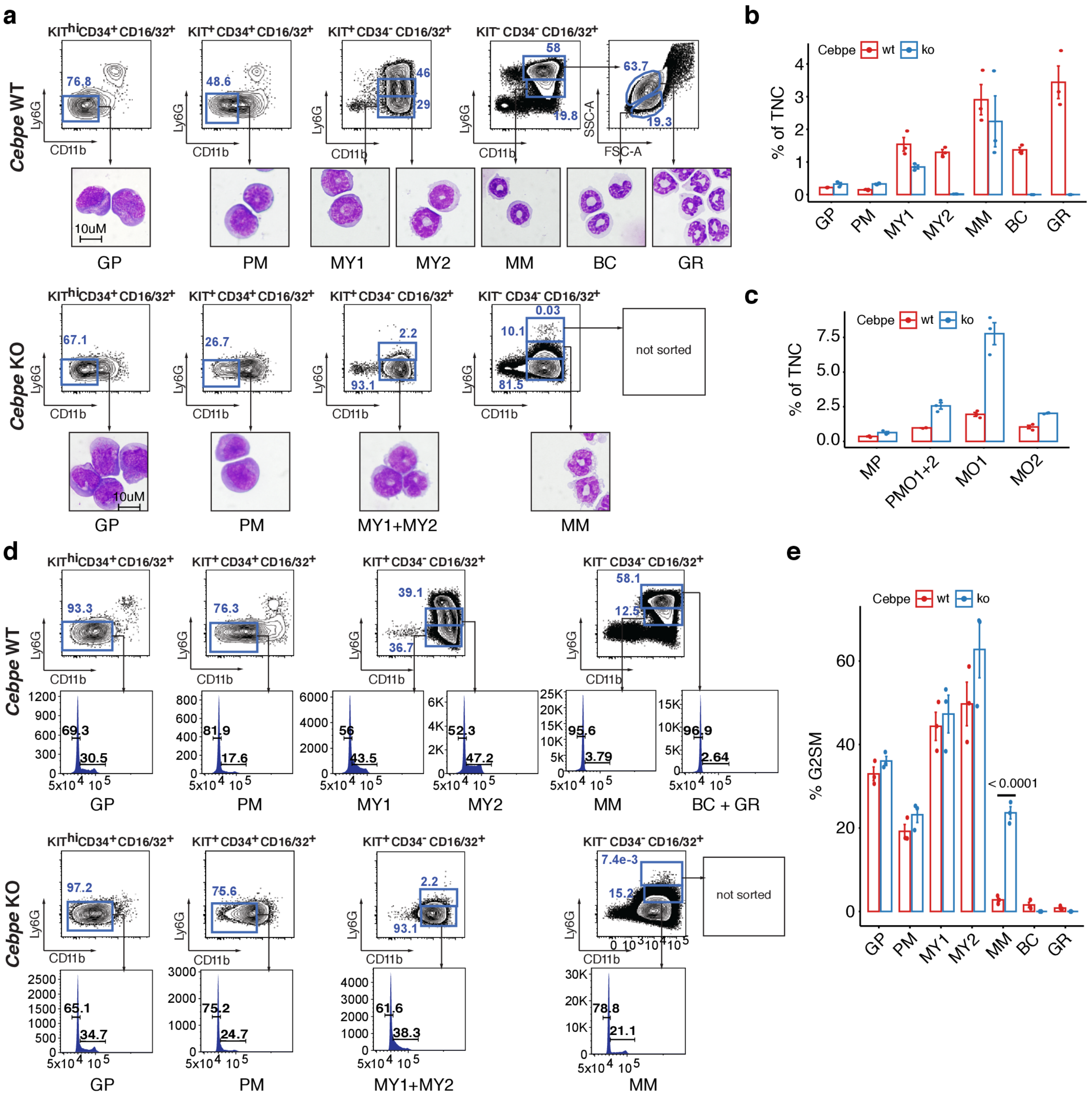

Supplementary Figure 1: Comparison of late granulocytic and monocytic differentiation hierarchies in *Cebpe* WT and *Cebpe* KO mice. a Immunophenotype, morphology, and **b-c** frequencies of murine bone marrow (BM) populations representing developmental stages of late granulocytic and monocytic differentiation in *Cebpe* WT and *Cebpe* KO mice (N=3 biological replicates, mean, whiskers represent the standard error). **d** Cell cycle analyses, and **e** frequencies of murine bone BM populations in G2SM phase in *Cebpe* WT and *Cebpe* KO mice (N=3 biological replicates, mean, whiskers represent the standard error). A two-way Student's t-test was used to detect statistical significance between groups and report the p-value. Source data are provided as a Source Data file.

Abbreviations: a-e *Granulocytic differentiation hierarchy*: Granulocyte progenitors (GP), promyelocytess (PM), early and late myelocytes (MY1 MY2), metamyelocyte (MM), band cells (BC), and fully mature granulocyte (GR). Total nuclear cells (TNCs). **c** *Monocytic differentiation hierarchy*: Monocyte progenitors (MP), early and late promonocytes (PMO1, PMO2), and early and late monocytes (MO1, MO2).

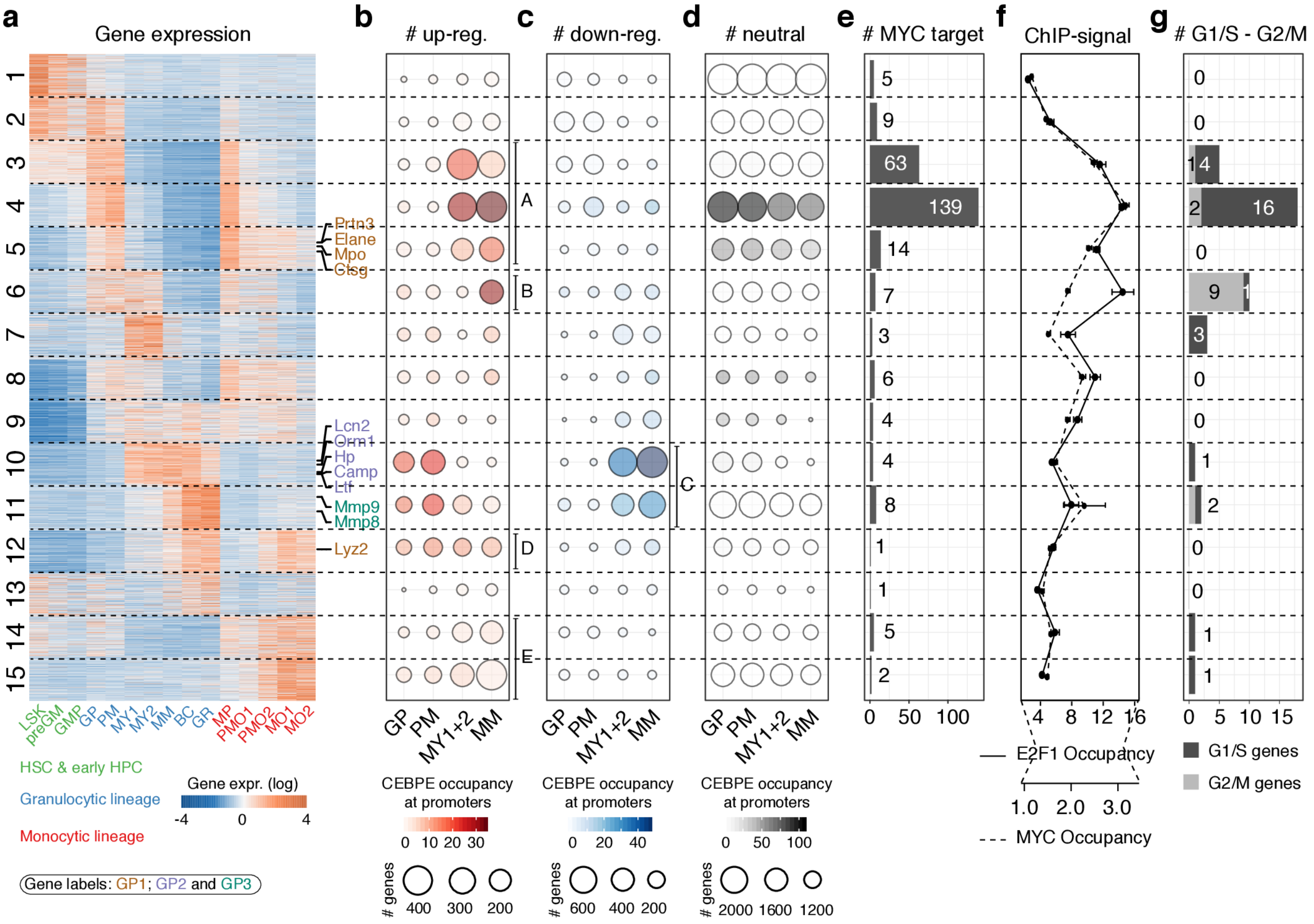

Supplementary Figure 2: Hierarchical cluster analysis of differentially expressed genes during early and late granulocytic and monocytic differentiation. a Heatmap of 15 gene clusters representing a total of 22,215 genes expressed during early and late granulocytic and monocytic differentiation (>5 reads in at least two cell populations). Clusters 5, 10 and 11 included sequentially expressed primary (brown), secondary (violet), and tertiary (green) granule proteins (GP1, GP2, GP3), respectively. **b-d** Bubble plots representing the number of genes in each cluster that are **(b)** up-regulated, **(c)** down-regulated, or **(d)** unaltered (neutral) in *Cebpe* KO vs. *Cebpe* WT GPs, PMs, MY1+2s, and MMs. The colors of the bubbles represent the enrichment levels of CEBPE binding at the promoters of up-regulated genes in each cluster as compared to the background (i.e. CEBPE binding levels at all gene promoters).

Subsets of the 15 clusters were merged into the following 5 major gene clusters represented in Figure 2 based on similarity, to empower subsequent functional analyses: Clusters 3, 4 and 5 were merged into cluster A(GMP) - genes expressed during early granulocytic and monocytic differentiation (i.e., in LSKs, preGMs, GMPs, GPs, PMs, and MPs); cluster 6 matches cluster B(MY) - genes transiently expressed in MY1s and MY2s; clusters 10 and 11 were merged into cluster C(GR) - genes terminally up-regulated in MMs, BCs, and GRs during late granulocytic differentiation; cluster 12 matches cluster D(GR+MO) - genes terminally up-regulated during both late granulocytic and monocytic differentiation; clusters 14 and 15 were merged into cluster E(MO) - genes up-regulated exclusively during monocytic differentiation in PMO1s, PMO2s, MO1s, and MO2s. **e** Frequencies of known MYC target genes as defined by Perna et al. [1] in individual clusters. **f** E2F1 (solid line) and MYC (dashed line) occupancy at the promoters of genes from the 15 clusters (N=2310, 1752, 1928, 2306, 1476, 1349, 1075, 928, 890, 1433, 1973, 1208, 781, 1164, 1642). Shown are the mean ChIP-seq signals in ESCs along with the standard errors. **g** Frequencies of key G1/S or G2/M phase cell cycle genes as defined by Giotti et al. [2] in individual clusters. Source data are provided as a Source Data file.

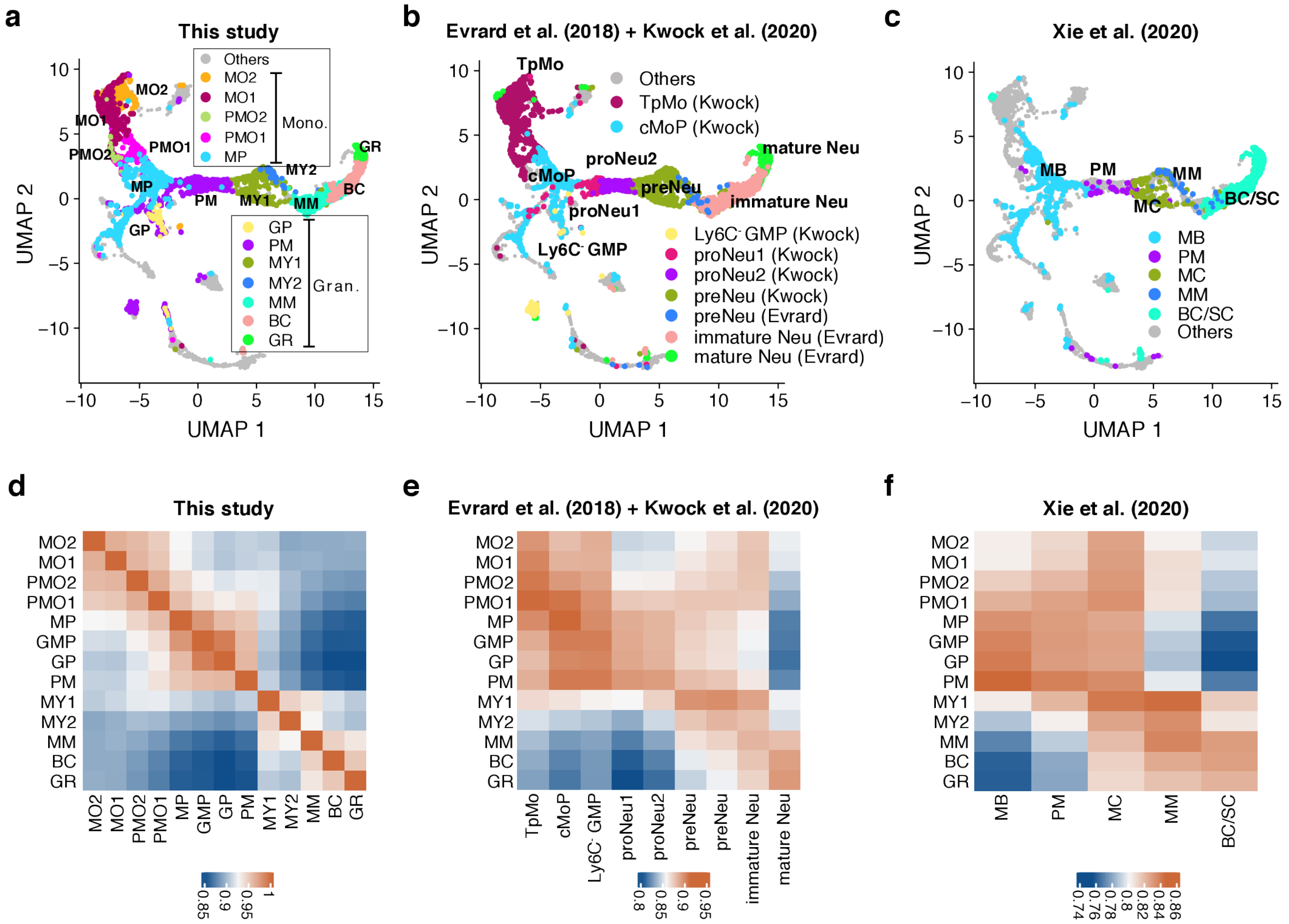

Supplementary Figure 3: Gene expression profiles of sorted BM populations were similar to those of previously reported BM populations. a-c UMAP depicting Seurat v4.0.1 integration of bone marrow single cell RNA-seq datasets by Kwock et al. (SmartSeq2) and Tabula Muris (10x) [3–5]. Single cells were annotated using bulk gene expression profiles of **(a)** sorted BM populations from this study, **(b)** sorted BM populations reported by Evrard and Kwock et al., and **(c)** sorted BM populations reported by Xie et al. [3,6,7]. **d-f** Spearman correlation between the gene expression profile of BM populations from this study **(d)** with that from Evrard and Kwock et al. **(e)**, and from Xie et al. **(f).** Source data are provided as a Source Data file.

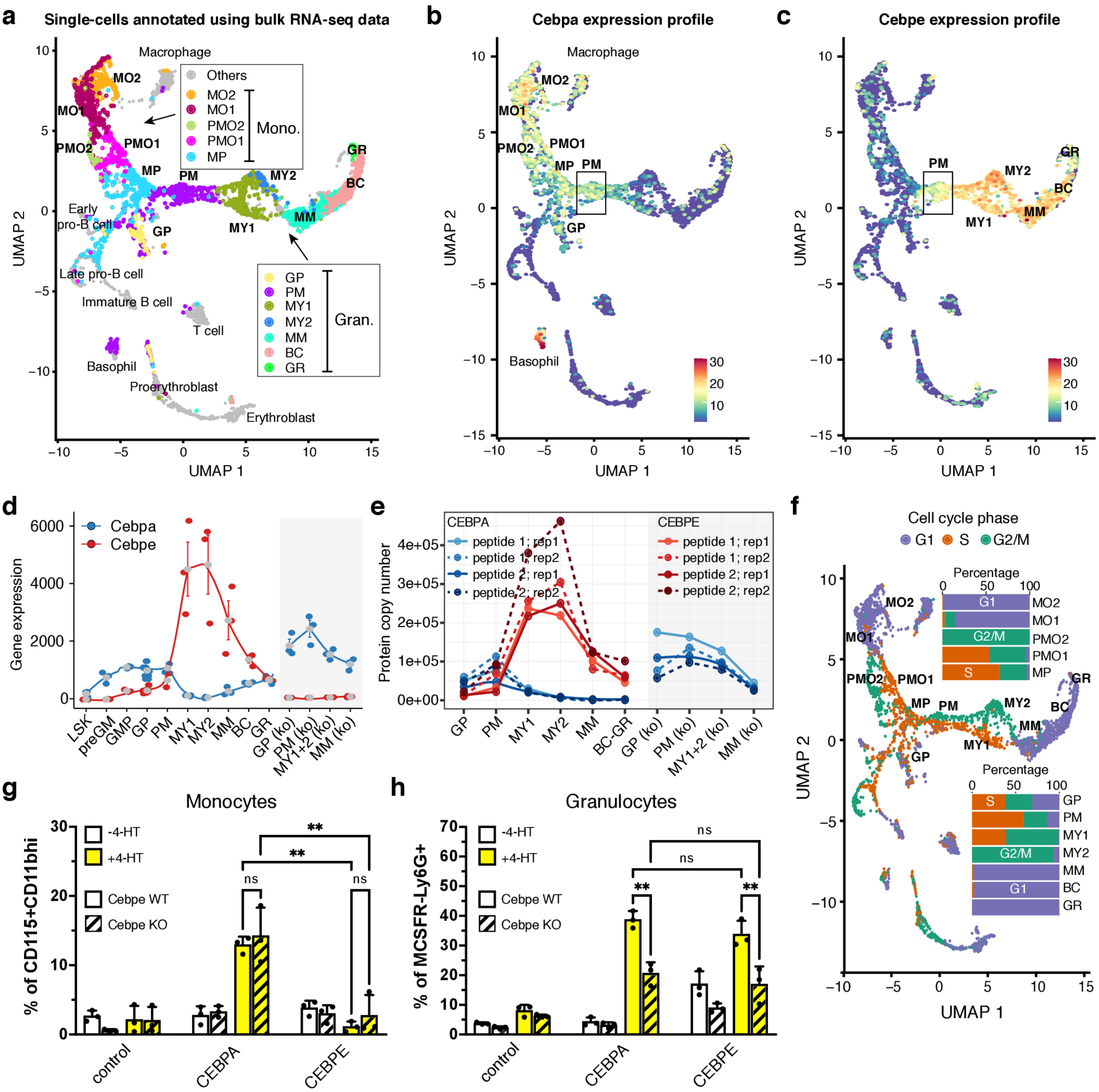

Supplementary Figure 4: BM populations exhibit sequential and almost exclusive expression of *Cebpa*/CEBPA and *Cebpe*/CEBPE at the RNA and protein level during granulocytic differentiation. a UMAP depicting Seurat v4.0.1 integration of bone marrow single cell RNA-seq datasets by Kwock et al. (SmartSeq2) and Tabula Muris (10x) [3–5]. Single cells were annotated using bulk gene expression profile of sorted BM populations from Figure 1. **b-c** UMAP depicting the expression profiles of *Cebpa* **(b)** and *Cebpe* **(c)**. **d** *Cebpa* and *Cebpe* gene expression profiles of sorted BM populations in *Cebpe* WT and KO mice (N=6 for the GP (WT) population, N=2 for the PM (WT) population and N=3 biological replicates for all other BM populations, mean, whiskers represent the standard error). **e** CEBPA and CEBPE protein expression profiles of sorted BM populations in *Cebpe* WT and KO mice (biological replicates). **f** UMAP illustrating the cell cycle states of individual cells as determined using Seurat v4.0.1 [5]. Also shown are the percentage of single cells annotated to specific sorted BM population that are in G1, S and G2/M cell cycle phases. **g-h** Immature KIT+ BM cells of *Cebpe* WT and KO mice were transduced with retroviral vectors expressing CEBPA-WT-ER^{TM}, CEBPE-WT-ER^{TM}, or control vector. After 4-HT induction of CEBPA and CEBPE activities, the frequencies of immunophenotypic monocytes (i.e. CD115+CD11b+ cells) **(g)** and granulocytes (i.e.CD115-Ly6G+ cells) **(h)** were assessed by flow cytometry analyses. While CEBPA induced both granulocytic and monocytic differentiation, CEBPE only induced granulocytic differentiation (N=3 biological replicates, mean, whiskers represent the standard error). One-way ANOVA was used to detect statistical significance between groups **(g-h)**. Tukey's multiple comparisons test was used for multiple comparisons between 2 groups and p-values were reported as follows: ns: not significant; *: p< 0.05; **: p<0.01. Source data are provided as a Source Data file.

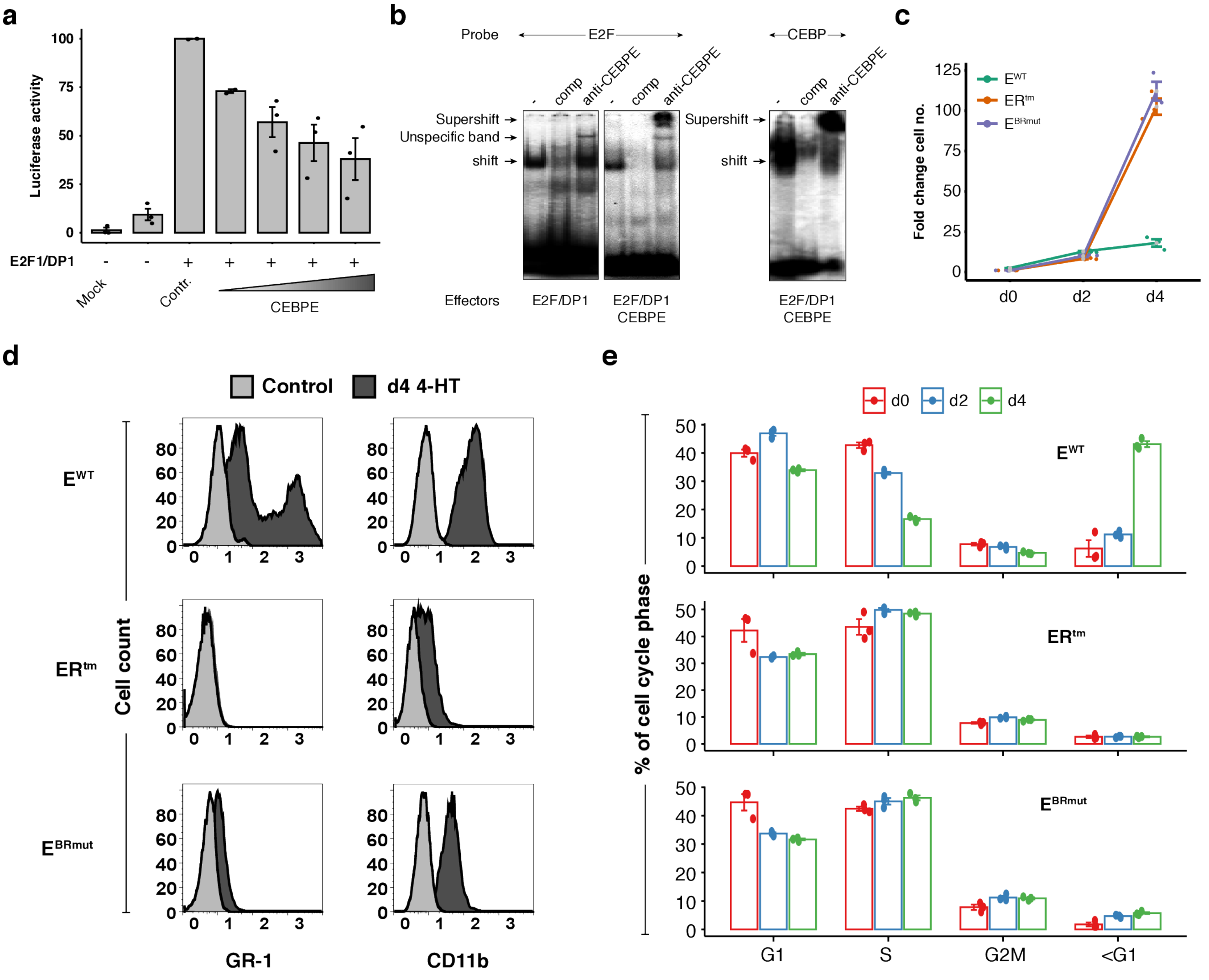

Supplementary Figure 5: CEBPE represses E2F and promotes cell cycle exit and differentiation. **a** Luciferase reporter assay: Q2bn fibroblasts were transiently transfected with a pE2Fx6-TATA-LUC reporter, pCMV-E2F1, pCMV-DP1, pcDNA3-CEBPE-WT expression vectors and a pCMVneoBam control vector as indicated (n=3 technical replicates). Empty pcDNA3 vector was used to adjust the total amount of DNA per dish, and the beta galactosidase reporter, pRSV-βGAL, was used to normalize luciferase activity. After 24h, cells were assayed for luciferase reporter gene expression (mean, whiskers represent the standard error). **b** EMSA assays: Q2bn fibroblasts were transiently transfected with indicated pCMV-E2F1, pCMV-DP1, pcDNA3-CEBPE-WT expression vectors. After 24h nuclear extracts were subjected to EMSA assays (N=1 each) using indicated radioactively labeled E2F and CEBPE probes with or without non-labeled probes and anti-CEBPE antibody. E2F/DP1 complexes bound to the E2F probe (shift, right panel) and demonstrated a supershift in the presence of CEBPE and anti-CEBPE antibody. CEBPE bound to the CEBP probe (shift, left panel) and demonstrated a supershift in the presence of anti-CEBPE antibody. **c-e** Analyses of 32DCl3 cell lines constitutively expressing an ER^{TM} control protein and the fusion proteins CEBPE-WT-ER^{TM} (E^{WT}) and CEBPE-BRmut-ER^{TM} (E^{BRmut} = basic region mutation that abrogates CEBP binding to E2F, Porse et al.)[8] were treated with 4-HT (N=3 technical replicates). **(c)** Cells were enumerated following 2 and 4 days of 4-HT induction, and the fold-change of cell numbers were calculated relative to day 0 (N=3 technical replicates, mean, whiskers represent the standard error). **(d)** The 32DCl3 cell lines were induced for 4 days with 4-HT and expression of the granulocytic differentiation markers GR1 and CD11b analyzed by flow cytometry following staining with anti-GR1, anti-CD11b and control antibodies. **(e)** The 32DCl3 cell lines were stained with propidium iodide and cell cycle phases analyzed by flow cytometry before and after 2 and 4 days of 4-HT induction (N=3 technical replicates, mean, whiskers represent the standard error)**.** Source data are provided as a Source Data file.

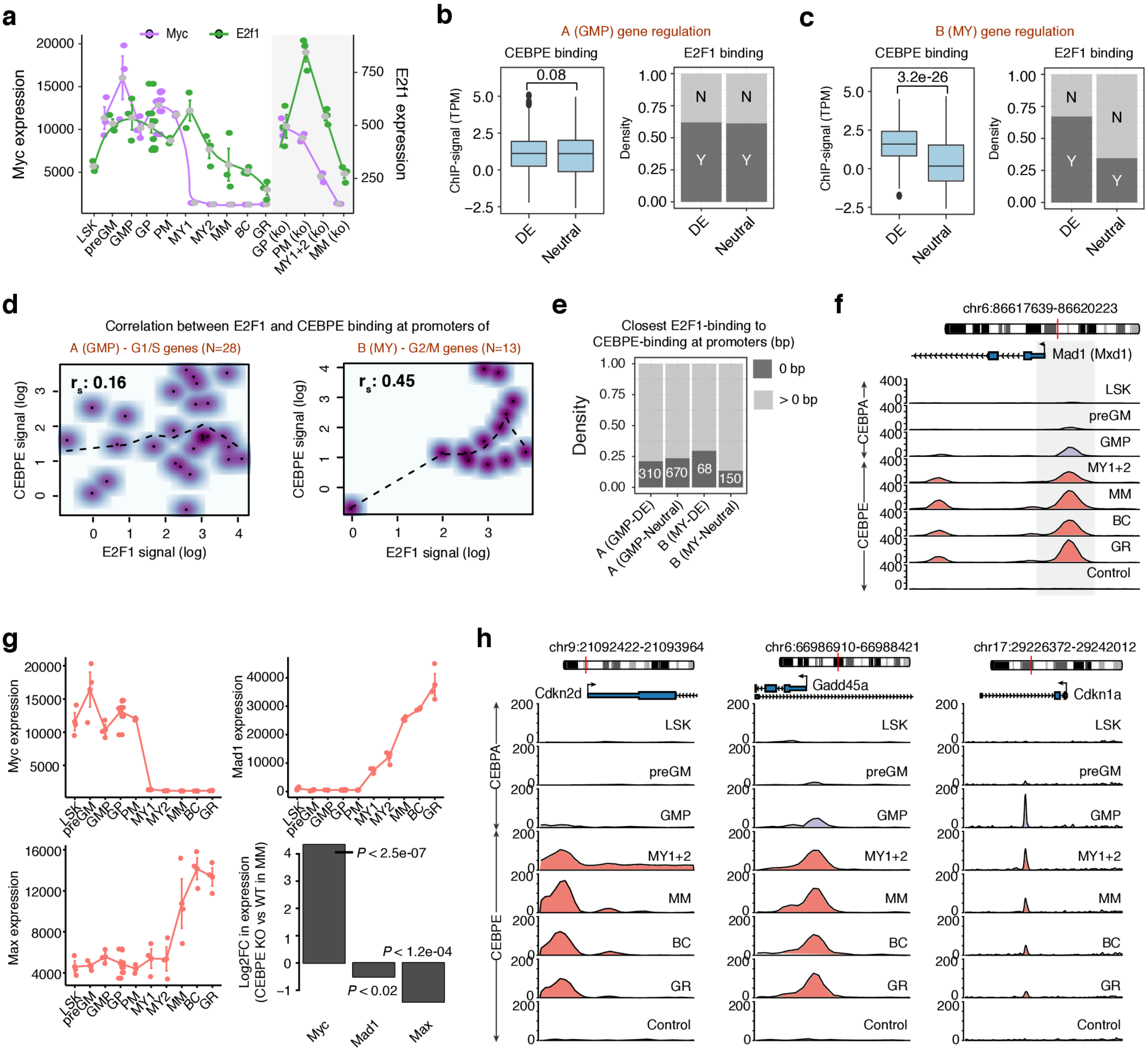

Supplementary Figure 6: CEBPE coordinates the down-regulation of G1/S genes by E2F1-mediated repression of *Myc*. a *Myc* and *E2f1* expression profiles in *Cebpe* WT and *Cebpe* KO during early and late granulocytic differentiation (N=3 (LSK), 3 (preGM), 3 (GMP), 6 (GP), 2 (PM), 3 (MY1), 3 (MY2), 3 (MM), 3 (BC), 3 (GR), 3 (GP KO), 3 (PM KO), 3 (MY1+2 KO), 3 (MM KO) biological replicates) (mean, whiskers represent the standard error). **b-c** CEBPE binding signal at promoters of differentially expressed (DE) and neutral genes of cluster A(GMP) (N=1475 DE and 2856 neutral) **(b)** and cluster B(MY) (N=231 DE and 1118 neutral) **(c)**, including fractions of DE and neutral genes that are bound by E2F1 at their promoters (center line, median; box limits, upper and lower quartiles; whiskers, 1.5x interquartile range; points, outliers) (one-sided Wilcoxon-test). **d** Spearman's rank correlation between E2F and CEBPE binding at the promoters of cluster A(GMP) and cluster B(MY) genes, including G1/S and G2/M phase genes, respectively. **e** Fractions of DE and neutral genes in *Cebpe* KO vs. *Cebpe* WT mice that are co-bound by CEBPE and E2F1 (i.e. 0 bp binding distance) or only bound by CEBPE (>0 bp binding distance) among cluster A(GMP) and cluster B(MY) genes. **f** Genome browser view of CEBPA and CEBPE binding in proximity to the *Mad* (*Mxd1*) gene promoter. **g** Expression profile of *Myc*, *Mad* and *Max* during early and late granulocytic differentiation (N=3 (LSK), 3 (preGM), 3 (GMP), 6 (GP), 2 (PM), 3 (MY1), 3 (MY2), 3 (MM), 3 (BC), 3 (GR) biological replicates), and their log2 fold-change (log2FC) expression in *Cebpe* KO vs. *Cebpe* WT MMs (mean, whiskers represent the standard error) (P-values represent significance level of differential expression from DESeq2). **h** Genome browser view of CEBPA and CEBPE binding to cis-regulatory elements of the CDK4, CDK2 and CDK1 inhibitors (*Cdkn2d*, *Cdkn1b, Gadd45a)* which are all up-regulated during late granulocytic differentiation. Source data are provided as a Source Data file.

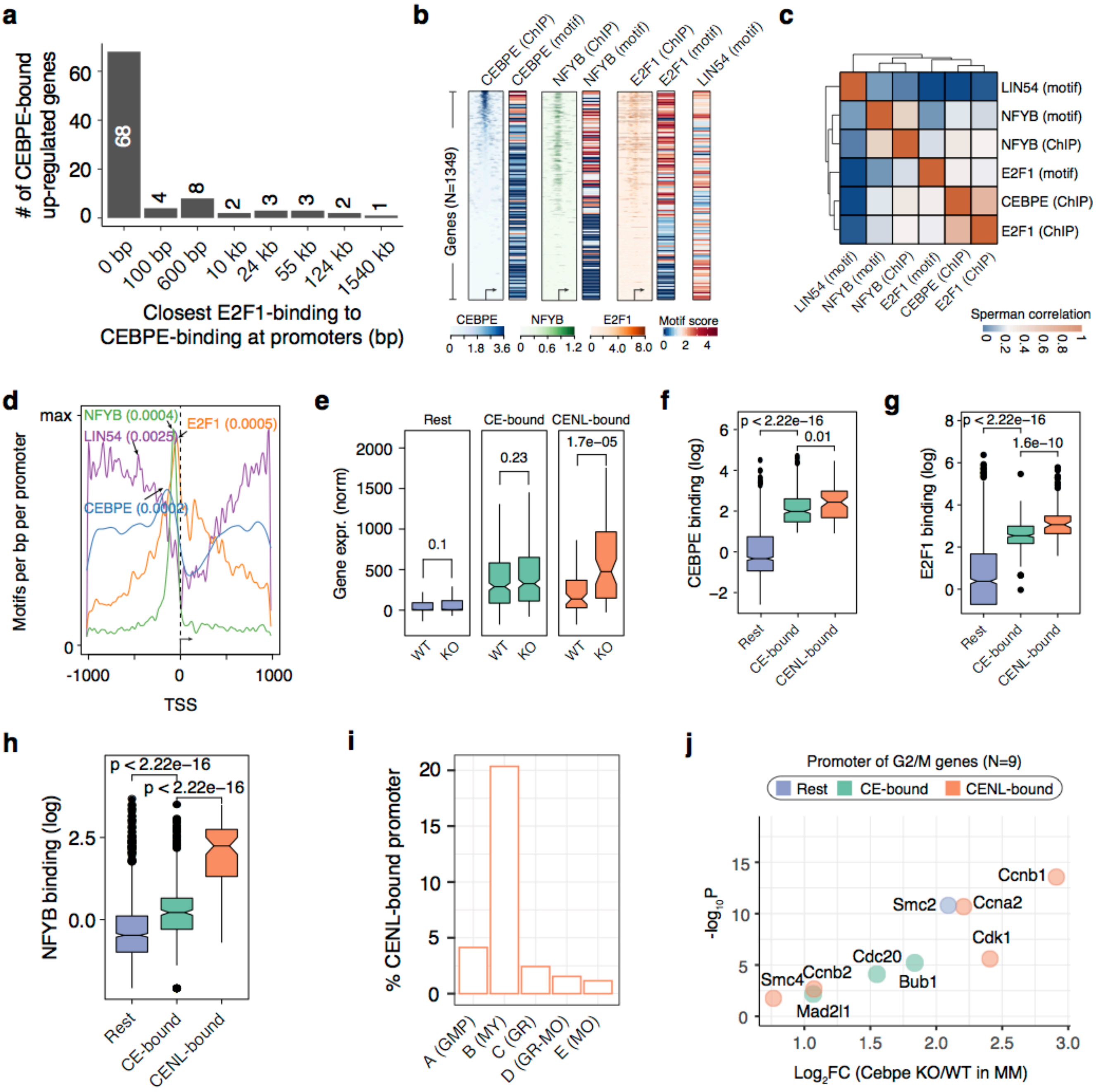

Supplementary Figure 7: Cooperative binding of CEBPE, NFYB and E2F1 at promoters of G2/M phase genes. a Frequencies of up-regulated genes (*Cebpe* KO vs. *Cebpe* WT) from cluster B(MY) classified based on the proximity of CEBPE and E2F1 binding at their promoters. **b** CEBPE, NFYB, E2F1 and LIN54 binding signals at the promoters of cluster B(MY) genes, which include the majority of G2/M phase genes. A high correlation of binding was inferred based on ChIP-seq and sequence motif scores for CEBPE, NFYB and E2F1. Please note that NFYB and E2F1 ChIP-seq data were derived from embryonic stem cells (ESCs). **c** Spearman´s correlation for the binding affinities of CEBPE, NFYB, E2F1 and LIN54 at cluster B(MY) genes, including the majority of G2/M phase genes. Except for LIN54, binding affinities for CEBPE, NFYB and E2F1 are highly correlated suggesting cooperative binding of these three TFs. **d** Relative positions of sequence binding motifs of all four TFs to the TSS of cluster B(MY) genes. **e** Absolute changes of expression of cluster B(MY) genes subdivided into gene subclasses based on combinatorial binding of CEBPE, NFYB, E2F1 and LIN54 at their promoters (CEBPE=C, E2F1=E, NFYB=N, LIN54=L). CENL subclass (orange): Binding of all four TFs (N=76), CE subclass (green): Binding of C and E with or without additional binding of either N or L (N=315), Rest (blue) (N=958): Binding of other TF combinations of C, E, N, L or none these (see also Figure 5c) (two-sided Wilcoxon-test) **f-h** Binding signal for CEBPE **(f)**, E2F1 **(g)**, and NFYB **(h)** at the promoters of CENL (N=76), CE (N=315) and the Rest (N=958) gene subclasses (center line, median; box limits, upper and lower quartiles; whiskers, 1.5x interquartile range; points, outliers) (one-sided Wilcoxon-test). **i** Percentage of genes of the five gene clusters depicted in Figure 2 that are bound by all four TFs (CENL-bound genes). **j** Bubble plot depicting the log2FC of

expression for 9 G2/M phase genes as a function of their significance level upon CEBPE KO. Bubbles are colored based on the binding of the four TFs (CEBPE, E2F1, NFYB, LIN54). Five out of the nine G2/M phase genes are bound by all the four TFs at their promoters. Source data are provided as a Source Data file.

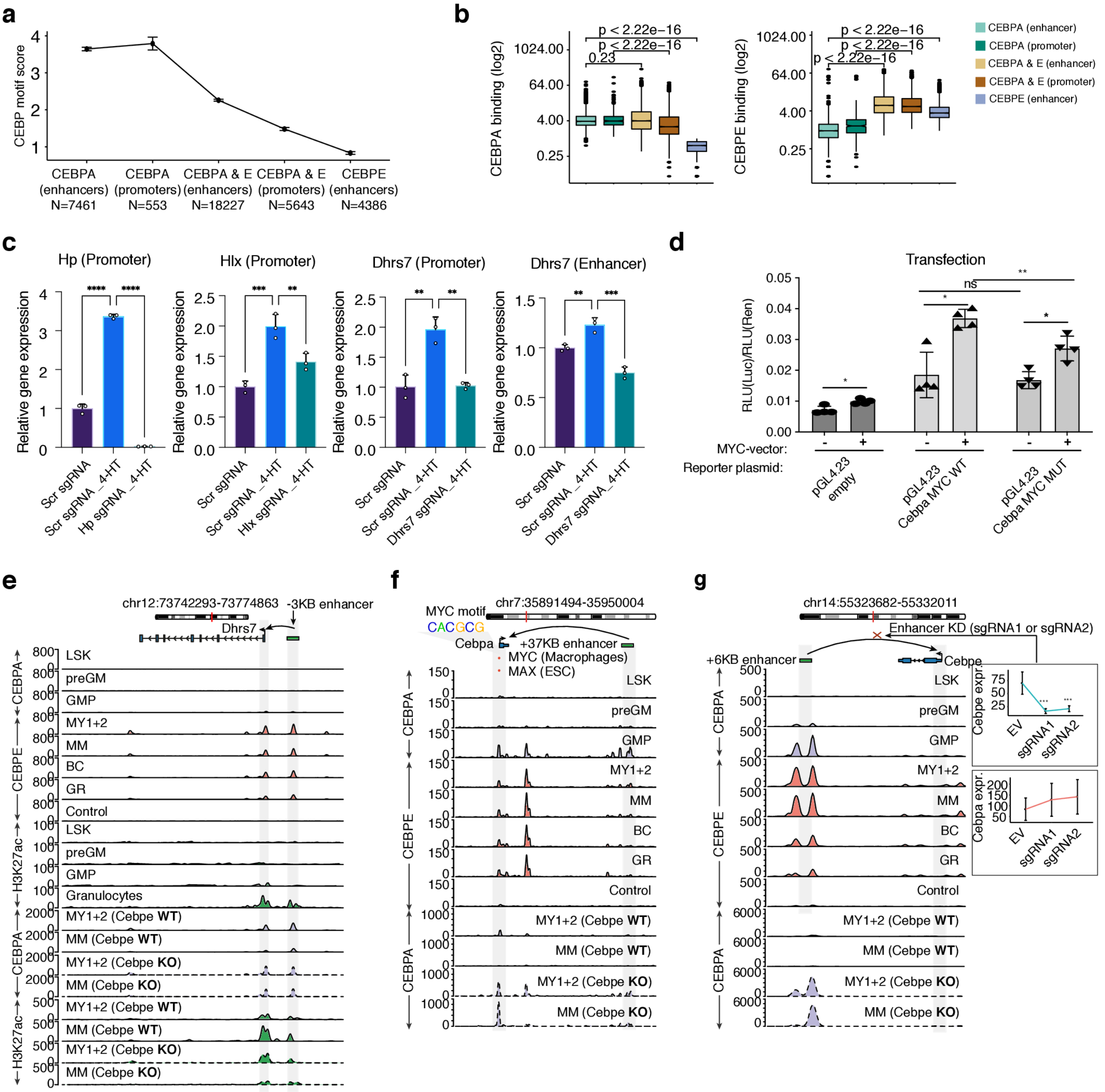

Supplementary Figure 8: CEBPA and CEBPE binding dynamics to cis-regulatory elements and their reciprocal regulation of expression during early and late granulocytic differentiation. a Median CEBP motif score at five classes of CEBPA and/or CEBPE bound regions (as defined in Figure 6d) (mean, whiskers represent the standard error). A higher score reflects CEBP motif assisted binding of CEBPA or CEBPE. **b** Levels of CEBPA and CEBPE binding at the five CEBPA and/or CEBPE bound region classes (as defined in Figure 6d) (center line, median; box limits, upper and lower quartiles; whiskers, 1.5x interquartile range; points, outliers) (Wilcoxon-test, one-sided). **c** CRISPRi KD of putative promoters and enhancers of genes regulated by CEBPE *in vivo* using the 32Dcl3-*CEBPE-ER*^{TM} cell line following induction of CEBPE activity and differentiation by 4-hydroxy-tamoxifen (4-HT). Two putative promoter-regulated genes (*Hp*, *Hlx*) and one enhancer-/promoter-regulated gene (*Dhrs7*) that were upregulated after 4-HT treatment exhibited decreased expression after CRISPRi KD of their CEBPE promoter (*Hp*, *Dhrs7*, *Hlx)* and enhancer (*Dhrs7*) sites. Two-way ANOVA was used to detect statistical significance between groups (N=3 biological replicates). Tukey's multiple comparisons test was used for multiple comparisons between 2 groups and p-values were reported as follows: ns: not significant; *: p< 0.05; **: p<0.01; ***: p< 0.001; ****: p<0.0001. **d** Validation of putative *Myc* binding site of the *Cebpa* promoter using a luciferase reporter assay: HEK293 cells were co-transfected with a *Myc* expression vector and reporter constructs harboring a 57-base pair *Cebpa* promoter fragment with and without its MYC binding E-box (MYC WT vs MYC MUT). A two-way Student's t-test was used to detect statistical

significance between groups (N=4 technical replicates) and p-values were reported as follows: ns: not significant; *: p< 0.05; **: p<0.01. **e** Genome browser view of the *Dhrs7* gene and its -3KB enhancer. Shown are the CEBPA, CEBPE and H3K27ac levels during granulocytic differentiation in the indicated BM populations of *Cebpe* WT and KO mice. **f** Genome browser view of the *Cebpa* gene and its +37KB enhancer. Unlike the *Cebpe* enhancer, the *Cebpa* enhancer is only bound by CEBPA during granulocytic differentiation and exhibits MYC and MAX binding to its promoter (i.e. ChIP-seq data from macrophages and embryonic stem cells (ESCs)) suggesting that *Cebpa* expression might be regulated by activating MYC/MAX dimers and repressive MAD/MAX dimers during early and late granulocytic differentiation, respectively. Also shown are the CEBPA binding levels in MY1+2 and MM populations derived from *Cebpe* WT and KO mice. **g** Genome browser view of the *Cebpe* gene and its +6KB enhancer, which is initially bound by CEBPA and then by CEBPE in indicated BM populations of *Cebpe* WT and KO mice. Line blots to the left demonstrate expression of *Cebpe* and *Cebpa* in response to sgRNAs disruption of the +6KB *Cebpe* enhancer activity as reported by Shyamsunder et al. [9]. Source data are provided as a Source Data file.

Supplementary References

1. Perna, D. *et al.* Genome-wide mapping of Myc binding and gene regulation in serum-stimulated fibroblasts. *Oncogene* 31, 1695–1709 (2012).

2. Giotti, B. *et al.* Assembly of a parts list of the human mitotic cell cycle machinery. *Journal of molecular cell biology* 11, 703–718 (2019).

3. Kwok, I. *et al.* Combinatorial Single-Cell Analyses of Granulocyte-Monocyte Progenitor Heterogeneity Reveals an Early Uni-potent Neutrophil Progenitor. *Immunity* 53, 303-318.e5 (2020).

4. Schaum, N. *et al.* Single-cell transcriptomics of 20 mouse organs creates a Tabula Muris. *Nature* 562, 367–372 (2018).

5. Hao, Y. *et al.* Integrated analysis of multimodal single-cell data. *Cell* 184, 3573-3587.e29 (2021).

6. Evrard, M. *et al.* Developmental Analysis of Bone Marrow Neutrophils Reveals Populations Specialized in Expansion, Trafficking, and Effector Functions. *Immunity* 48, 364-379.e8 (2018).

7. Xie, X. *et al.* Single-cell transcriptome profiling reveals neutrophil heterogeneity in homeostasis and infection. *Nat Immunol* 21, 1119–1133 (2020).

8. Porse, B. T. *et al.* E2F repression by C/EBPalpha is required for adipogenesis and granulopoiesis in vivo. *Cell* 107, 247–258 (2001).

9. Shyamsunder, P. *et al.* Identification of a novel enhancer of CEBPE essential for granulocytic differentiation. *Blood* 133, 2507–2517 (2019).
