# Supplementary material for: Transcription factor-driven coordination of cell cycle exit and lineage-specification in vivo during granulocytic differentiation: In memoriam Professor Niels Borregaard
Source: Nat Commun. 2022 Jun 23;13:3595. doi: 10.1038/s41467-022-31332-1 (PMC9225994; doi:10.1038/s41467-022-31332-1)
Supplement: Supplementary file 3 — Description of Additional Supplementary Files [file 41467_2022_31332_MOESM3_ESM.pdf]

Description of Additional Supplementary Files

Supplementary Data 1: Expression of genes analyzed (N=22215), their cluster information and Transcription factor binding and motif score at their promoters.

Supplementary Data 2: Genomic coordinates of CEBPA and/or CEBPE bound regions, their putative target genes and CEBP motif scores.
