# Supplementary material for: Transcription factor-driven coordination of cell cycle exit and lineage-specification in vivo during granulocytic differentiation: In memoriam Professor Niels Borregaard
Source: Nat Commun. 2022 Jun 23;13:3595. doi: 10.1038/s41467-022-31332-1 (PMC9225994; doi:10.1038/s41467-022-31332-1)
Supplement: Supplementary file 6 — Reporting Summary [file 41467_2022_31332_MOESM6_ESM.pdf]

nature research

Corresponding author(s): Bo Porse and Kim Theilgaard-Monch
Last updated by author(s): May 6, 2022

Reporting Summary

Nature Research wishes to improve the reproducibility of the work that we publish. This form provides structure for consistency and transparency in reporting. For further information on Nature Research policies, see our Editorial Policies and the Editorial Policy Checklist.

Statistics

For all statistical analyses, confirm that the following items are present in the figure legend, table legend, main text, or Methods section.

n/a	Confirmed	
☐	☒	The exact sample size (*n*) for each experimental group/condition, given as a discrete number and unit of measurement
☒	☐	A statement on whether measurements were taken from distinct samples or whether the same sample was measured repeatedly
☐	☒	The statistical test(s) used AND whether they are one- or two-sided<br>*Only common tests should be described solely by name; describe more complex techniques in the Methods section.*
☒	☐	A description of all covariates tested
☐	☒	A description of any assumptions or corrections, such as tests of normality and adjustment for multiple comparisons
☐	☒	A full description of the statistical parameters including central tendency (e.g. means) or other basic estimates (e.g. regression coefficient) AND variation (e.g. standard deviation) or associated estimates of uncertainty (e.g. confidence intervals)
☐	☒	For null hypothesis testing, the test statistic (e.g. *F*, *t*, *r*) with confidence intervals, effect sizes, degrees of freedom and *P* value noted<br>*Give P values as exact values whenever suitable.*
☒	☐	For Bayesian analysis, information on the choice of priors and Markov chain Monte Carlo settings
☒	☐	For hierarchical and complex designs, identification of the appropriate level for tests and full reporting of outcomes
☐	☒	Estimates of effect sizes (e.g. Cohen's *d*, Pearson's *r*), indicating how they were calculated

Our web collection on statistics for biologists contains articles on many of the points above.

Software and code

Policy information about availability of computer code

Data collection	Sequencing of CEBPE ChIP-seq libraries was performed on the Nextseq500, 50 bp single-end. Sequencing of RNA-seq libraries was performed on the HiSeq2500, 75 bp paired-end. Sequencing of CEBPA and H3K27ac ChIP-seq libraries was performed on the Nextseq500, 41 bp paired-end, Raw reads were aligned to the mouse genome (mm9 genome assembly) with bowtie v2 2.3.4.3. Primary reads from each bam were normalized to reads-per-million and bigWig tracks visualized using Gviz v1.18.2.
Data analysis	STAR v020201, Rsubread v1.24.2, clusterprofiler v3.2.14, Bowtie2 v2.3.4.3, Macs2 v2.1.0.20151222, IDR, Caret v6.0-84, Gviz v1.18.2, ngs.plot v2.61, homer v4.10.4, R v3.4.4, Seurat v4.0.1, SingleR v1.8.0, ComplexHeatmap v2.1.0; FlowJo v10.8.1<br><br>Data analysis script: https://github.com/porseLab/Cebpe

For manuscripts utilizing custom algorithms or software that are central to the research but not yet described in published literature, software must be made available to editors and reviewers. We strongly encourage code deposition in a community repository (e.g. GitHub). See the Nature Research guidelines for submitting code & software for further information.

Data

Policy information about availability of data

All manuscripts must include a data availability statement. This statement should provide the following information, where applicable:

- Accession codes, unique identifiers, or web links for publicly available datasets
- A list of figures that have associated raw data
- A description of any restrictions on data availability

We have deposited our data in the GEO repository. If interested the reviewers can access it via the following:
Go to https://www.ncbi.nlm.nih.gov/geo/query/acc.cgi?acc=GSE159430

Enter token qdqlmmoavbatfin into the box

Publicly available data sources used in this study:
GSE89767 (RNA-seq LSK, preGM, GMP, GP and MP),
GSE11431, GSE56839 (MYC, E2F1 and NFYB ChIP-seq data),
GSE151630 (Kwock et al. Single cell data),
GSE109467 (Evrard et al. Single cell data),
GSE137538 (Xie et al. Single cell data),
The Tabula Muris consortium single cell data v3.

Field-specific reporting

Please select the one below that is the best fit for your research. If you are not sure, read the appropriate sections before making your selection.

[x] Life sciences [ ] Behavioural & social sciences [ ] Ecological, evolutionary & environmental sciences

For a reference copy of the document with all sections, see nature.com/documents/nr-reporting-summary-flat.pdf

Life sciences study design

All studies must disclose on these points even when the disclosure is negative.

Sample size	Not relevant for this study - as this is not a clinical trial. A minimum of 2 replicates from each of the 14 cell populations were subjected to RNA-seq and ChIP-seq experiments. The exact no of replicates is given in the relevant figure legends and the material and methods section.
Data exclusions	Data are not excluded from analysis.
Replication	Reproducibility of biologicial replicates was evaluated and confirmed by plotting PCA plots and as shown in Figure 1D. All replicates clustered together. Various ChIP-seq experiments with different Abs also demonstrated reproducibility of biological replicates.
Randomization	Not relevant for this study - as this is not a clinical trial.
Blinding	Not relevant for this study - as this is not a clinical trial.

Reporting for specific materials, systems and methods

We require information from authors about some types of materials, experimental systems and methods used in many studies. Here, indicate whether each material, system or method listed is relevant to your study. If you are not sure if a list item applies to your research, read the appropriate section before selecting a response.

Materials & experimental systems

n/a	Involved in the study	
[ ]	[x]	Antibodies
[x]	[ ]	Eukaryotic cell lines
[x]	[ ]	Palaeontology and archaeology
[ ]	[x]	Animals and other organisms
[x]	[ ]	Human research participants
[x]	[ ]	Clinical data
[x]	[ ]	Dual use research of concern

Methods

n/a	Involved in the study	
[ ]	[x]	ChIP-seq
[ ]	[x]	Flow cytometry
[x]	[ ]	MRI-based neuroimaging

Antibodies

Antibodies used

Antibodies used for LSK, preGMP, and GMP FACS analyses and sorting:

CD150-APC (cat. no. 115910, clone TCF15-12F12.2; BioLegend, San Diego, California, USA),
CD41-FITC (cat. no. 11-041-82, clone MWReg30; eBiosciences/ ThermoFisher Scientific, Waltham, Massachusetts, USA),
CD105-PE or CD105-PE-Cy7 (cat. no. 12-1051-82, 25-1051-82, clone Mj7/18; eBiosciences/ ThermoFisher Scientific),
CD115-PE (cat. no. 12-1152-82, clone AFS98; eBiosciences/ ThermoFisher Scientific),
Sca1-PerCP-Cy5.5 (cat. no. 45-5981-82, clone D7; eBiosciences/ ThermoFisher Scientific),
CD117-A780 (cat. no. 47-1171-82, clone 2B8; eBiosciences/ ThermoFisher Scientific),
FcgRII/III-A700 (cat. no. 56-0151-82, clone 93, eBiosciences/ ThermoFisher Scientific).

Lineage cocktail consisting of the following PE-Cy5 conjugated antibodies:
Ter119-PE-Cy5 (cat. no 116210, clone Ter119, BioLegend),

	Gr1-PE-Cy5 (cat. no. 15-5931-82, clone RB6-8C5; eBiosciences/ ThermoFisher Scientific),<br>B220-PE-Cy5 (cat. no. 15-0452-82, clone RA3-6B2; eBiosciences/ ThermoFisher Scientific),<br>CD3e-PE-Cy5 (cat. no. 15-0031-82, clone 145-2C11; eBiosciences/ ThermoFisher Scientific),<br>Mac1-PE-Cy5 (cat. no. 101210, clone M1/70, BioLegend).<br><br>anti-CD117-MoAb microbeads (cat. no. 130-091-224, Miltenyi Biotec, Bergisch Gladbach, DE).<br><br>Antibodies used for analyses and sorting of BM populations representing sequential developmental stages of late granulocytic (GP, PM, MY1, MY2, MM, BC, GR) and late monocytic (MP, PMO1, PMO2, MO1, MO2) differentiation hierarchies:<br><br>CD34-FITC (cat. no. 11-0341-82, clone RAM34; eBiosciences/ ThermoFisher Scientific),<br>CD115-PE (cat. no. 12-1051-82, clone AFS98, BioLegend),<br>Ter119-PE-Cy5 (cat. no 116210, clone Ter119, BioLegend),<br>B220-PE-Cy5 (cat. no. 15-0452-82, clone RA3-6B2; eBiosciences/ ThermoFisher Scientific),<br>CD3e-PE-Cy5 (cat. no. 15-0031-82, clone 145-2C11; eBiosciences/ ThermoFisher Scientific),<br>NK-1-PE-Cy5 (cat. no. 108716, clone PK136, BioLegend),<br>Sca1-PerCP-Cy5.5 (cat. no. 45-5981-82, clone D7; eBiosciences/ ThermoFisher Scientific),<br>CD11b-PE-Cy7 (cat. no. 25-0112-82, clone M1/70; eBiosciences/ ThermoFisher Scientific),<br>Ly6G-APC-CY7 (cat. no. 127624, clone 1A8, BioLegend).<br><br>Antibodies used for ChIP-seq experiments:<br><br>CEBPE (cat. no. sc-25770, H-75, Santa Cruz Biotechnology discontinued),<br>CEBPA (cat. no. sc-61, clone 14AA, Santa Cruz Biotechnology, discontinued),<br>H3K27ac (cat. no. ab4729, Abcam, Cambridge, UK), and<br>irrelevant IgG control (cat. no. sc-2027, Santa Cruz Biotechnology).
Validation	All antibodies provided by BioLegend/eBiosciences were validated by flow cytometry on mouse bone marrow cells.<br>Antibodies provided by Santa Cruz Biotechnology were validated by Western blotting on whole cell lysates of K-562 cells.

Animals and other organisms

Policy information about studies involving animals; ARRIVE guidelines recommended for reporting animal research

Laboratory animals	Cebpe KO and littermates of both sexes were used. In addition, female C57BL/6 WT mice were used. All mice used were 10-12 weeks old; Yamanaka, R. et al. Impaired granulopoiesis, myelodysplasia, and early lethality in CCAAT/enhancer binding protein epsilon-deficient mice. Proceedings of the National Academy of Sciences 94, 13187–13192 (1997).
Wild animals	No wild animals were used in this study.
Field-collected samples	No field-collected samples were used in this study.
Ethics oversight	The study was approved under the license no. 2012-15-2935-01 by the Danish Animal Research Inspectorate

Note that full information on the approval of the study protocol must also be provided in the manuscript.

ChIP-seq

Data deposition

☒ Confirm that both raw and final processed data have been deposited in a public database such as GEO.

☒ Confirm that you have deposited or provided access to graph files (e.g. BED files) for the called peaks.

Data access links<br>*May remain private before publication.*	https://www.ncbi.nlm.nih.gov/geo/query/acc.cgi?acc=GSE159430
Files in database submission	ChIP-seq and RNA-seq (fastq and bigWig) files.<br><br>GSM4828867 cebpe_lpmmy_Rep1<br>GSM4828868 cebpe_lpmmy_Rep2<br>GSM4828869 cebpe_mm_Rep1<br>GSM4828870 cebpe_mm_Rep2<br>GSM4828871 cebpe_bc_Rep1<br>GSM4828872 cebpe_bc_Rep2<br>GSM4828873 cebpe_pmn_Rep1<br>GSM4828874 cebpe_pmn_Rep2<br>GSM4828875 cebpe_ko_control_Rep1<br>GSM4829652 gp_wt_rep1 [RNA-Seq]<br>GSM4829653 gp_wt_rep2 [RNA-Seq]<br>GSM4829654 gp_wt_rep3 [RNA-Seq]

GSM4829655 gp_ko_rep1 [RNA-Seq]
GSM4829656 gp_ko_rep2 [RNA-Seq]
GSM4829657 gp_ko_rep3 [RNA-Seq]
GSM4829658 mp_wt_rep1 [RNA-Seq]
GSM4829659 mp_wt_rep2 [RNA-Seq]
GSM4829660 mp_wt_rep3 [RNA-Seq]
GSM4829661 pm_wt_rep1 [RNA-Seq]
GSM4829662 pm_wt_rep2 [RNA-Seq]
GSM4829663 pm_ko_rep1 [RNA-Seq]
GSM4829664 pm_ko_rep2 [RNA-Seq]
GSM4829665 pm_ko_rep3 [RNA-Seq]
GSM4829666 pmo1_wt_rep1 [RNA-Seq]
GSM4829667 pmo1_wt_rep2 [RNA-Seq]
GSM4829668 pmo2_wt_rep1 [RNA-Seq]
GSM4829669 pmo2_wt_rep2 [RNA-Seq]
GSM4829670 pmo2_wt_rep3 [RNA-Seq]
GSM4829671 mo1_wt_rep1 [RNA-Seq]
GSM4829672 mo1_wt_rep2 [RNA-Seq]
GSM4829673 mo2_wt_rep1 [RNA-Seq]
GSM4829674 mo2_wt_rep2 [RNA-Seq]
GSM4829675 mo2_wt_rep3 [RNA-Seq]
GSM4829676 my1_wt_rep1 [RNA-Seq]
GSM4829677 my1_wt_rep2 [RNA-Seq]
GSM4829678 my1_wt_rep3 [RNA-Seq]
GSM5704730 cebpa_my12_wt_Rep1
GSM5704731 cebpa_my12_wt_Rep2
GSM5704732 cebpa_my12_ko_Rep1
GSM5704733 cebpa_my12_ko_Rep2
GSM5704734 cebpa_mm_wt_Rep1
GSM5704735 cebpa_mm_wt_Rep2
GSM5704736 cebpa_mm_ko_Rep1
GSM5704737 cebpa_mm_ko_Rep2
GSM5704738 h3k27ac_my12_wt_Rep1
GSM5704739 h3k27ac_my12_wt_Rep2
GSM5704740 h3k27ac_my12_ko_Rep1
GSM5704741 h3k27ac_my12_ko_Rep2
GSM5704742 h3k27ac_mm_wt_Rep1
GSM5704743 h3k27ac_mm_wt_Rep2
GSM5704744 h3k27ac_mm_ko_Rep1
GSM5704745 h3k27ac_mm_ko_Rep2
GSM5704746 input_my12_wt_Rep1
GSM5704747 input_my12_ko_Rep1
GSM5704748 input_mm_wt_Rep1
GSM5704749 input_mm_ko_Rep1
GSM4829679 my1+2_ko_rep1 [RNA-Seq]
GSM4829680 my1+2_ko_rep2 [RNA-Seq]
GSM4829681 my1+2_ko_rep3 [RNA-Seq]
GSM4829682 my2_wt_rep1 [RNA-Seq]
GSM4829683 my2_wt_rep2 [RNA-Seq]
GSM4829684 my2_wt_rep3 [RNA-Seq]
GSM4829685 mm_wt_rep1 [RNA-Seq]
GSM4829686 mm_wt_rep2 [RNA-Seq]
GSM4829687 mm_wt_rep3 [RNA-Seq]
GSM4829688 mm_ko_rep1 [RNA-Seq]
GSM4829689 mm_ko_rep2 [RNA-Seq]
GSM4829690 mm_ko_rep3 [RNA-Seq]
GSM4829691 bc_wt_rep1 [RNA-Seq]
GSM4829692 bc_wt_rep2 [RNA-Seq]
GSM4829693 bc_wt_rep3 [RNA-Seq]
GSM4829694 gr_wt_rep1 [RNA-Seq]
GSM4829695 gr_wt_rep2 [RNA-Seq]
GSM4829696 gr_wt_rep3 [RNA-Seq]

Genome browser session
(e.g. UCSC)

bigWig files corresponding to each ChIP-seq and RNA-seq experiment is available at https://www.ncbi.nlm.nih.gov/geo/query/acc.cgi?acc=GSE159430 for visualization in the Genome Browser (UCSC or Gviz).

Methodology

Replicates

Two replicates each for CEBPE ChIP-seq experiments on MY1+2, MM, BC, GR cell populations.

Sequencing depth

Sample #reads #mapped read_length type
cebpe_bc_Rep1 53373698 22517421 50 single-end
cebpe_bc_Rep2 65489892 26117644 50 single-end
cebpe_control_Rep1 43953717 11769777 50 single-end
cebpe_my1+2_Rep1 41631502 30065755 50 single-end
cebpe_my1+2_Rep2 54878330 23846149 50 single-end
cebpe_mm_Rep1 47899465 21944037 50 single-end
cebpe_mm_Rep2 28822343 9490425 50 single-end
cebpe_gr_Rep1 32621378 22099394 50 single-end
cebpe_gr_Rep2 48481136 33541798 50 single-end
cebpa_mm_wt_Rep1 6406598 2612327 41 paired-end
cebpa_mm_wt_Rep2 9674487 5036581 41 paired-end
cebpa_mm_ko_Rep1 5665019 1020890 41 paired-end
cebpa_mm_ko_Rep2 12169595 8306841 41 paired-end
cebpa_my12_wt_Rep1 3441276 869375 41 paired-end
cebpa_my12_wt_Rep2 8184492 1923339 41 paired-end
cebpa_my12_ko_Rep1 5133051 2161010 41 paired-end
cebpa_my12_ko_Rep2 9372363 6709053 41 paired-end
h3k27ac_mm_wt_Rep1 10283465 5671890 41 paired-end
h3k27ac_mm_wt_Rep2 25565791 19477523 41 paired-end
h3k27ac_mm_ko_Rep1 23064900 15080272 41 paired-end
h3k27ac_mm_ko_Rep2 24891024 17522528 41 paired-end
h3k27ac_my12_wt_Rep1 19068010 13907073 41 paired-end
h3k27ac_my12_wt_Rep2 21881447 16673185 41 paired-end
h3k27ac_my12_ko_Rep1 16457799 11722152 41 paired-end
h3k27ac_my12_ko_Rep2 21465351 16330533 41 paired-end
input_mm_wt_Rep1 26301376 19635199 41 paired-end
input_mm_ko_Rep1 29894228 20768467 41 paired-end
input_my12_wt_Rep1 23407342 17162507 41 paired-end
input_my12_ko_Rep1 24306863 17206870 41 paired-end

Antibodies

Chromatin was incubated with an antibody targeting CEBPE (H-75, Santa Cruz), CEBPA (clone 14AA, Santa Cruz Biotechnology, discontinued), H3K27ac (ab4729, Abcam, Cambridge, UK), and irrelevant IgG control (sc-2027, Santa Cruz Biotechnologies).

Peak calling parameters

Genomic regions enriched for CEBPE in MY1+2s, MMs, BCs and PMNs, were determined by peak calling using MACS2 (parameters: -g mm -p 1e-3 --nomodel --extsize SHIFT_SIZE. Here, SHIFT_SIZE is determined using macs2 predictd command). To increase the specificity of the enriched regions, we used a control sample (CEBPE ChIP in Cebpe KO cells). Irreproducible Discovery Rate (IDR) was used at a false discovery rate of 0.05 to filter out irreproducible regions between the two replicates.

Data quality

Sample #peaks (FDR < 0.05)
cebpe_MY1+2 28264
cebpe_MM 23119
cebpe_BC 19149
cebpe_GR 15514

Software

Macs2 v2.1.0.20151222 and IDR

Flow Cytometry

Plots

Confirm that:

- [x] The axis labels state the marker and fluorochrome used (e.g. CD4-FITC).
- [x] The axis scales are clearly visible. Include numbers along axes only for bottom left plot of group (a 'group' is an analysis of identical markers).
- [x] All plots are contour plots with outliers or pseudocolor plots.
- [x] A numerical value for number of cells or percentage (with statistics) is provided.

Methodology

Sample preparation

Murine BM cells were collected from tibiae, femura, and ilia of 8-10 weeks old mice. For FACS analyses or cell sorting both cristae, tibiae, and femora from mice were crushed in a mortar containing 10 mL PBS+2 %FCS, flushed 3 times, and cells filtered through a 70µm filter into a 15-mL tube. BM cells were stained were stained with fluorochrome-conjugated antibodies for 30min on ice. After incubation, BM cells were washed and resuspended at 10 106/ml in PBS/3%FCS and

	7AAD (1ug/ml, Invitrogen, Carlsbad, California, USA) prior to flowcytometry analysis and cell sorting, and only 7AAD- cells were included in the analyses and sorts.
Instrument	BD LSR II or BD ARIA III flow cytometers (BD Biosciences, San Jose, CA, USA) were used for all experiments.
Software	FlowJo analysis software (Version 10.1, TreeStar Inc., San Carlos, CA, USA) was used for analysis of all flow cytometry data. Gates defining positive and negative populations for specific markers were set according to fluorescence-minus-one (FMO) controls stained with isotype-matched control antibodies.
Cell population abundance	The mean abundance of populations among total nuclear BM cells were as follows: LSK wt - 0.08%, Cebpe KO - 0.09 %; preGMP wt - 0.35%, Cebpe KO - 0.55 %; GMP wt - 0.3%, Cebpe KO - 0.7 %. The abundance of all other populations are given in detail in Suppl. Figure S1B and S1C. Examples of relative population frequencies are given in the FACS plots of Figure 1 and Suppl. Figure S1. Purities of sorted BM populations was defined by FACS re-analyses and were a least >90%.
Gating strategy	The gating strategy is depicted in detail i Fig 1. FSC/SSC levels indicating the treshold boundaries between "positive" and "negative" are depicted in Fig. 1b lower panel to the right (FSC/SSC plot).

☒ Tick this box to confirm that a figure exemplifying the gating strategy is provided in the Supplementary Information.
